# UV Resistance of bacteria from the Kenyan Marine cyanobacterium *Moorea producens*

**DOI:** 10.1002/mbo3.697

**Published:** 2018-08-19

**Authors:** Thomas Dzeha, Constance Nyiro, Dimitris Kardasopoulos, David Mburu, Joseph Mwafaida, Michael J. Hall, J. Grant Burgess

**Affiliations:** ^1^ Department of Chemical Science and Technology Technical University of Kenya Nairobi Kenya; ^2^ Department of Biological Sciences Pwani University Kilifi Kenya; ^3^ School of Natural and Environmental Sciences Newcastle University Newcastle UK; ^4^ School of Chemistry Newcastle University Newcastle UK

**Keywords:** *Bacillus licheniformis*, bacteria, benzophenone‐3, first‐order kinetics, *Moorea producens*, UV resistance

## Abstract

UV resistance of bacteria isolated from the marine cyanobacterium *Moorea producens* has not been observed previously, findings which highlight how unsafe germicidal UV irradiation for sterilization of air, food, and water could be. Further, UV resistance of *Bacillus licheniformis* is being observed for the first time. This study focused on bacteria isolated from the marine cyanobacterium *M. producens* collected off the Kenyan coast at Shimoni, Wasini, Kilifi, and Mida. UV irradiance of isolates (302 nm, 70 W/m^2^, 0–1 hr) established *B. licheniformis* as the most UV resistant strain, with the following order of taxon resistance: *Bacilli*> γ *proteobacteria* > *Actinobacteria*. UV resistance was independent of pigmentation. The maximum likelihood phylogenetic distance determined for both *B. licheniformis* and *Bacillus aerius* relative to *M. producens* CCAP 1446/4 was 2.0. Survival of *B. licheniformis* upon UV irradiance followed first‐order kinetics (*k* = 0.035/min, *R* ^2^ = 0.88). Addition of aqueous extracts (2, 10, 20 and 40 mg/ml) of this *B. licheniformis* strain on the less resistant *Marinobacterium stanieri* was not significant, however, the commercial sunscreen benzophenone‐3 (BP‐3) positive control and the time of irradiance were significant. Detection of bacteria on *M. producens* filaments stained with acridine orange confirmed its nonaxenic nature. Although the chemistry of UV resistance in cyanobacteria has been studied in depth revealing for example the role of mycosporine like amino acids (MAAs) in UV resistance less is known about how bacteria resist UV irradiation. This is of interest since cyanobacteria live in association with bacteria.

## 1 INTRODUCTION

The oceans are recipients of high solar radiation and an understanding of the adaptations of organisms therein toward the deleterious effects of UV radiation can aid the search for novel photoprotective agents. The vast majority of UV‐C radiation (100–290 nm) is filtered out by the Earth's atmosphere, while UV‐A (315–400 nm) and UV‐B (290–320 nm) wavelengths persist. UV‐B is the cause of 90% of all skin cancers (El‐Mahdy et al., 2008; Reagan‐Shaw, Breur, & Ahmad, 2006; Young, 2009). The less erythrogenic and carcinogenic UV‐A, mainly contributes to photoaging (Burke & Wei, 2009; Schreurs, Lanser, Seinen, & van der Burg, 2002).

Cyanobacteria fulfill vital ecological functions in the world's oceans, being important contributors to global carbon and nitrogen budgets. They are arguably the most successful group of microorganisms on earth, having existed for the last 3 billion years. They are genetically diverse; occupy a variety of niches including habitats across all latitudes, marine, and terrestrial ecosystems and are found in extreme environments such as hot springs, salt works, and hypersaline bays. In response to radiation, cyanobacteria utilize photoprotective compounds such as scytonemin and its derivatives, mycosporine‐like amino acids (MAAs) with high molar extinction coefficients to resist UV radiation (Gao & Garcia‐Pichel, 2011; Siezen, 2011; Torres et al., 2004). Mycosporines and MAAs are UV‐absorbing small molecules (*λ*_max_ = 310–360 nm) and are also synthesized by fungi and eukaryotic micro‐ and macroalgae (Bandaranayake, Bemis, & Bourne, 1996; Shick & Dunlap, 2002). Their success in preventing UV‐induced skin damage in vivo has led to their commercialization, for example in the products Helioguard 365 and Helionori sunscreens to protect against UV‐A (Balskus & Walsh, 2010; De la Coba et al., 2009; Siezen, 2011).

A sustainable supply of such sunscreen molecules and other bioactive cyanobacterial compounds could be achieved through aquaculture, chemical synthesis or by recombinant biosynthesis from a source organism. However, obtaining sunscreens through aquaculture is not currently economically feasible as cyanobacteria have a complex circadian rhythm compared with bacteria and it is expensive to grow them (Kondo & Ishiura, 2000). Low enantiomeric excess (e.e) yields and refractory problems associated with chemical synthesis are further limitations to realising sustainability.

Fossil evidence predates 440 Ma for mutually beneficial functional and metabolically interactive associations between cyanobacteria and heterotrophic bacteria (Tomescu, Honegger, & Rothwell, 2008). Bacteria routinely associate with cyanobacteria for buoyancy, organic carbon, nitrogen, and sheath nutrients. In return cyanobacteria benefit from increased photosynthesis arising from removal of oxygen sequestered by bacteria during symbiotic growth (Paerl, Pinckney, & Steppe, 2000). However, much research on bacteria–cyanobacteria interactions has been conducted, virtually none has focused on whether bacteria can contribute to the UV resistance of their host cyanobacterium (Hube, Heyduck‐Söller, & Fischer, 2009; Salomon, Janson, & Granéli, 2003; Tuomainen, Hietanen, Kuparinen, Martikainen, & Servomaa, 2006). It is also possible that cyanobacteria provide UV protection to symbiotic strains as well as symbionts enhancing the UV protection of cyanobacteria. Bacteria are easy to grow and could offer sustainable yields of photoprotective agents for commercial application.

This study aimed to investigate the nonaxenic nature of Kenyan *M. producens* and whether or not bacteria from *M. producens* isolates showed UV resistance. The phylogenetic relationships between the most UV resistant bacteria and their host cyanobacterium are presented. Investigations were also made to establish if aqueous extracts of *Bacillus licheniformis* strain BLC‐01 (KC660142) could impart UV protection on *Marinobacterium stanieri* strain MARIS‐02 (KC660134) cultures. This is the first study to investigate bacteria from a marine cyanobacterium for their UV resistance properties and to quantitatively follow the kinetics of UV resistance in an isolate of *B. licheniformis*.

## 2 MATERIALS AND METHODS

### 2.1 Collection and identification of *M. producens*

Bacteria in this study were isolated from samples of the filamentous marine cyanobacteria *M. producens* identified previously (Davies‐Coleman et al., 2003). *Moorea producens* specimens were collected from Kilifi (039.785˚E to 039.835˚E) and Mida Creek (039.99505º to 039.96600ºE) on the North Coast; and at Shimoni (039.36565ºE to 039.36696ºE) and Wasini (039.35906ºE to 039.35942ºE) on the South Coast of Kenya in 2011. The choice of location was based on the ubiquitous availability of the cyanobacteria and on geographical positioning. *M. producens* specimens were handpicked from a 100 m transect along the shore line to a water level of nearly 0.5 m from the shore. *M. producens* samples were thereafter placed in sterile polythene bags and transported to the laboratory at Pwani University, Kilifi for storage at 4°C prior to workup. A second collection of *M. producens* at Shimoni was also made in 2012 and the specimens were similarly treated before transportation to the United Kingdom for analysis.

**Figure 1:**
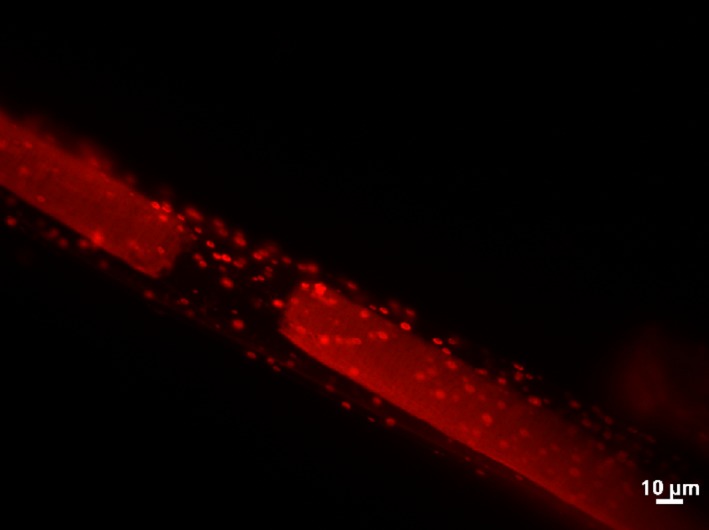
Acridine orange stained bacterial cells on the surface of a live *Moorea producens* filament

During workup in the laboratory, *M. producens* was treated with cycloheximide (5 mg/L, overnight) to reduce contaminating eukaryotic cells, protozoa, and fungi. It was then rinsed several times with filtered sterile seawater (12 times, 45 μl) and left overnight in phosphate poor autoclaved seawater. For detachment of filaments, the cyanobacterium was submerged in phosphate buffered saline (PBS, pH 7.4), filaments were rinsed with sterile water, pooled together and aliquots weighed to provide sufficient biomass for microscopy, DNA extraction, and genome sequencing. Filaments were stained with acridine orange and observed, using a Leica DMRB microscope with a Micropublisher digital camera (Figure**** 1).

### 2.2 Genomic isolation and identification of bacterial isolates

Bacterial isolates were streaked onto marine agar 2216 (Difco) for strain isolation. Pure isolates were obtained after successive streaking. Bacterial isolates were cultured in marine broth 2216 (50 ml, Difco) to generate biomass for DNA extraction. Cultures were incubated (28°C) with continuous shaking for 3–5 days after which they were harvested by centrifugation. DNA was extracted, using standard phenol–chloroform procedures (Sambrook & Russell, 2001).

The PureLink^R^ genomic DNA kit from Invitrogen was used according to manufacturer's instructions. Specifically, bacterial cells (200 mg) were treated with lysis buffer (1 ml) and incubated (65°C, 30 min). Following centrifugation (10,000 ×g, 5 min), 0.5 ml of the resulting supernatant was treated with genomic‐binding buffer (0.5 ml), centrifuged (10,000 rpm, 5 min) and the supernatant passed through a DNA‐binding column. The column was washed with Genomic wash buffer (1 ml) and 3 times with 1 ml 75% ethanol prior to dry spinning. DNA was eluted and collected from the column with 0.2 TE buffer.

The polymerase chain reaction (PCR) product of the 16S rDNA was obtained by reacting a mixture containing 2 × PCR buffer biomix (20 μl, BIOLINE^®^), 1 μl of primer 9F (5′‐GAGTTTGATCCTGGCTCAG‐3′), and 1 μl of primer 1492R (5′‐ TACGGYTACCTTGTTACGACTT‐3′). The final volume of the PCR mixture was adjusted to 50 μl by adding sterile Milli Q water. Thermal cycling was performed with a TECHNE Touchgene (USA) thermal cycler. Samples were subjected to an initial denaturation step (95°C, 5 min; 95°C, 30 s) followed by annealing (40°C, 1 min). The thermal profile used was 30 cycles consisting of 1 min of primer annealing at 55°C, 1 min of extension at 72°C and 1 min of denaturation at 95°C. A final extension step consisting of 10 min at 72°C was also included. PCR products were detected by agarose gel electrophoresis (GIBCO BRL, USA) and were visualized by UV fluorescence after ethidium bromide staining. The PCR products generated by the 9F and 1492R primers were approximately 1 kb in size and were purified using the PureLink^R^ genomic DNA kit from Invitrogen as specified by the manufacturer and sequenced bidirectionally with the primers 9F and 1492R from Sigma‐Aldrich (described above). Sequencing of the final DNA extracts was done by Genius Laboratories (UK). The 16S rDNA sequences determined in this study have been deposited with Genbank.

### 2.3 Phylogenetic analyses

**Figure 2:**
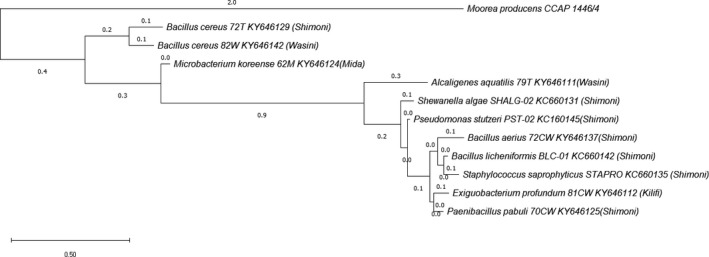
Phylogeny of UV resistant bacteria in relation to *Moorea producens* CCAP 1446/4

However, 16S rDNA Sequences of UV resistant bacteria were manipulated using ClustalW for pairwise and multiple alignments. Evolutionary analyses were conducted in MEGA6 (Tamura, Nei, & Kumar, 2004) in which the maximum composite likelihood model was used to estimate the evolutionary divergence between the sequences. Evolutionary distance was calculated using the neighbor‐joining method. Distances were obtained where the values represented the dissimilarity for each pairwise comparison (phylogenetic diversity). A phylogenetic tree was constructed by using the neighbour‐joining method and the tree reliability was determined by 2000 bootstrap replications for 95% reproducibility (Figure 2). Accession numbers of the determined sequences are reported in Table 1.

**Table 1:** UV resistant bacteria isolated from the filamentous marine cyanobacteria *Moorea producens* collected along the Kenya coast during 2011–2012

Bacterial strains	GenBank Accession number	Closest match in GenBank	% Similarity	Taxon	*T* (min)	Sample site
79T	KY646111	*Alcaligenes aquatilis* LL22996	97	β‐proteobacteria	45	Wasini
72CW	KY646137	*Bacillus aerius* strain *24K*	96	G+ Bacilli	45	Shimoni
72T	KY646129	*Bacillus cereus* ATCC 14579	94	G+ Bacilli	45	Shimoni
82W	KY646142	*Bacillus cereus* ATCC 14579	89	G+ Bacilli	45	Wasini
BLC‐01	KC660142	*B. licheniformis* NC_006270.3	99	G+ Bacilli	>60	Shimoni
81CW	KY646112	*Exiguobacterium profundum* strain *10C*	98	G+ Bacilli	45	Kilifi
62M	KY646124	*Microbacterium koreense* strain *JS3‐2*	97	Actinobacteria	45	Mida
70CW	KY646125	*Paenibacillus* pabuli strain NBRC *136*	99	G+ Bacilli	45	Shimoni
PST‐02	KC660145	*Pseudomonas stutzeri* NC_009434.1	99	γ‐proteobacteria	60	Shimoni
SHALG‐02	KC660131	*Shewanella algae* NZ_BALO0100009.1	99	γ‐proteobacteria	60	Shimoni
STAPRO	KC660135	*Staphylococcus saprophyticus* NC_007350.1	99	G+ Bacilli	45	Shimoni

*Note*. Sequence accession numbers of the isolated strains are shown in the second column, % similarity corresponds to previously reported organisms in the GenBank database; *T* in minutes represents the survival duration of the bacteria on UV exposure. The last column shows the sample site.

### 2.4 Short wavelength ultraviolet irradiance assays of bacteria isolates

Bacterial isolates from *M. producens* were streaked onto marine agar 2216 containing streptomycin sulfate antibiotic (50 μg/ml in H_2_O). The antibiotic was added to the media upon cooling to prevent the growth of fastidious bacteria. Plate covers were aseptically removed from the plates to eliminate errors arising from UV absorption by plastics. Bacterial isolates were screened for UV resistance (302 nm, 70 W/m^2^, 0–1 h), using agar‐based streak assays in petri dishes according to the method of Lin and Wang (Lin & Wang, 2001) with modifications. The coverless agar plates were turned upside down and exposed to a bottom‐up source of UV radiation (302 nm, 70 W/m^2^) in a gel documentation system UV/VIS radiometer (Genetic Research Instrumentation, Dunmow, UK). The radiometer consisted of a UVC 254 nm wavelength lamp that was established to have an intensity of 70 W/m^2^ at 302 nm by routine checking. The experiments in Kenya utilized a newly acquired custom‐built UV analysis cabinet (ACM 82307‐Delhi, India) with both UVA (365 nm) and UVC (254 nm) lamps. UV screens of the bacterial isolates utilized the UVC lamp at 254 nm. The surface on which the plates were irradiated was sterilized with 75% ethanol between experiments. UV exposed plates and controls without UV irradiance were incubated (37°C, 48 hr) after which the numbers of colonies resisting radiation were recorded.

For UV irradiance of planktonic bacteria in broth cultures, bacteria were inoculated in marine broth 2216. The inoculum was incubated using an orbital shaker (200 rpm, 37°C, 24 hr), harvested at the stationary phase (24 hr) and the optical density at 600 nm determined. Undiluted inoculum (500 μl) was spread onto marine agar plates for the UV irradiance assays. The populations of bacteria resisting UV irradiance exposure for 0, 15, 30, 45, and 60 min, respectively were used to determine the survival constants of the bacteria under UV irradiance.

### 2.5 UV irradiance aqueous extracts assay

*Bacillus licheniformis* strain BLC‐01 (KC660142) was cultured in marine broth media 2216 and incubated at 37°C. The broth culture, with an absorbance of 1.7 at OD 600 nm was passed through a 0.20 μm Millipore filter. The filtrate was centrifuged (3250*g*, 40 min) and freeze dried at −80°C to a fine powder. The powder was used to make concentrations of 2, 10, 20, and 40 mg/ml of aqueous extracts (AE) metabolites, respectively, in milli Q water. These AEs were added to cultures of the less UV resistant *Marinobacterium stanieri* strain MARIS‐02 (KC660134) and investigated for additive UV resistance effects on the bacterium.

For AE assays, *B. licheniformis* and *M. stanieri* isolates were cultured in marine broth media 2216 and incubated at 37°C. Absorbance values of cultures were recorded. Time of irradiance and concentration of extracts were the variables whereas 0 mg/ml of *B. licheniformis* metabolite extract was the negative control. The positive control comprised a 1 mg/ml solution of the commercial UV sunscreen Benzophenone‐3 (BP‐3) from Sigma Aldrich. The reconstituted culture was spread onto marine agar and irradiated. Experiments were performed in triplicate.

### 2.6 Analysis of UV‐irradiance data

During the 2012 collection of *M. producens* from Shimoni, kinetic studies for establishing the survival constant, k and an estimation of the time for *B. licheniformis* (most UV resistant bacteria) to die upon irradiance were done. Equations for the rate of death of the bacteria were constructed and these were based on the UV intensity, time, bandwidth, and fluence.

The survival population data of bacterial isolates upon UVB irradiation in this study suggested that UVB bacterial resistance was consistent with the following exponential decay equation:

(1) $$N_{t}=N_{0}e^{-\mathrm{KHT}},$$

where **N**_***t***_ and **N**_**0**_ are the populations at time **t** and at the initial time (control population) respectively, **k** is the survival constant (m/J), **H** is the total fluence (W m min^−1^) of the radiating lamp and **t** is the irradiance (exposure) time (min). The intensity was considered to represent the irradiance on a flat surface (petri dish) and was constant. However, the total fluence **H** is a function of the absolute transmittance **T**, the bandwidth of radiation **b** (nm), the fluence **H**_**0**_ (W m^−2^) and time ***t*** (min) **(**Mamane‐Gravetz, Linden, Cabaj, & Sommer, 2005)**.** Thus, we have the following:

(2) $$H\propto {\text{TbH}}_{0}t$$

The bandwidth **b** of the UV lamp used was 10 nm and transmittance was 6.3% based on OD_600_ values recorded. It was argued that at the lim **N**_**t**_ **→0**; that is, at the time when no bacteria survive, the population **N**_**t**_ approaches zero. Subsequently, solutions of the first order integral of Equation (1) resulted to the logarithmic equation for the survival constant, **k**.

(3) $$k={\left(H\left(e^{-t}-1\right)\right)}^{-1}\text{ln}\;N_{t}/N_{0}$$

and a solution of the time, **t:**

(4) $$t=\ln\;N_{0}/(1/\mathrm{kH}\;\mathrm{In}\;N_{t}$$

Using the log‐transformed version of Equation (3), data on surviving population **N**_**t**_, control population **N**_**0**_, time ***t*** and fluence **H** were used to estimate the survival constant of *B. licheniformis* exposed to UV radiation, for example but were applicable to the UV resistant bacteria in this study.

### 2.7 Statistical analysis

Most data were categorical and therefore analyzed using the nonparametric test statistic Pearson's Chi‐square Test for Count Data while applying continuity correction. In some cases, Fisher's exact test was used. The software R studio in R: A Language and Environment for Statistical Computing was used (Team RC, 2017). The Two‐way analysis of variance (ANOVA) was used to determine whether there were any statistically significant differences between the means of the independent groups under investigation.

## 3 RESULTS

### 3.1 Isolation and phylogenetic analysis of the UV resistant bacteria

A total of 88 bacterial strains were isolated from cyanobacteria from the four sites in 2011 and 20 additional strains isolated during the recollection at Shimoni in 2012. In 2011, the highest number of isolates was recorded at Shimoni and the least at Kilifi. The data showed that there was a significant difference in the morphological distribution of the bacteria among the different sites (Chi‐square value = 18.32, df = 6, *p = *0.005, N = 88). Some isolates were pigmented and others were not. Colors of the pigmented bacteria included orange, lemon yellow, deep yellow, pink, red and red brown.

Whereas there was no significant difference between pigmented and nonpigmented bacteria among different sites (*p = *0.08, Fishers Exact test, N = 88), pigmented bacteria were delineated according to their differences in color (Kruskal–Wallis value = 24.04, df = 13, *p = *0.031, N = 88). Microscopic identification classified the bacteria as being either single or clustered Cocci, single or chained *Bacilli* or Coccobacilli in shape.

In 2011, only 55 out of the initial 88 bacteria isolates were viable. These viable bacteria isolates that comprised 24 pigmented and 31 nonpigmented strains were exposed to UV irradiation at varied time intervals as a measure of inherent ability of pigmented and nonpigmented bacteria isolates to resist UV radiation. No significant difference between the ability of pigmented bacteria and nonpigmented bacteria to withstand or not withstand ultraviolet radiation (Fishers Exact test, *p = *0.067, *p = *0.21, *p = *0.66, N = 55) was observed. *Bacilli* exhibited the highest UV resistance (*t* = 60 min) with respect to time and β‐*proteobacteria* represented by *Alcaligenes aquatilis* (KY646111) showed medium resistance (*t* ≤ 45 min). The Actinobacterium, *Microbacterium koreense* (KY646124) was the least resistant of the isolates. During the recollection of *M. producens* at Shimoni in 2012 *B. licheniformis* (KC660143) and *Bacillus subtilis* (KC660144) representing Gram Positive Bacilli; and the Gram Negative *Shewanella algae* (KC660131) and *Pseudomonas stutzeri* (KC660145) respectively exhibited UV resistance out of 20 bacterial isolates. The nature of resistance was found to differ between Gram‐positive and Gram‐negative strains.

The list of UV resistant bacteria from this study is presented in Table 1. An examination of the phylogeny for UV resistant bacteria suggests that except for *Bacillus cereus* (KY646129, KY646142) and *Microbacteriun koreense* (KY646124) most of the bacteria may have evolved at the same time as *M. producens* CCAP 1446/4 albeit independently from their ancestors (Figure 2). Surprisingly, the evolutionary distances for *B. aerius* and *B. licheniformis* matched with that for *M. producens* CCAP 1446/4 at 2.0 on the phylogeny tree. It would be interesting to find out exactly the genetic traits common between *B. aerius, B. licheniformis* and *M. producens* CCAP 1446/4. It is also unclear why *B. cereus* (KY646129, KY646142) and *Microbacteriun koreense* (KY646124) that are UV resistant could have evolved earlier compared with the other bacterial species in this study.

### 3.2 Total fluence and transmittance as factors of irradiance

Cultures of surviving bacteria were observed 48 hr after incubation compared with controls of nonirradiated bacteria that grew after 24 hr. Overall, there was a marked increase in the size of colonies of UV resistant bacteria that was not observed with controls. Total fluence **H** (W m^−1^ min^−1^) was consistent with Equation (2) and increased linearly with fluence and time as predicted. Optical densities measured as **%T** were critical in the total fluence reaching the film of bacteria on the plate. Dilute cultures with an absorbance of 0.1 at 600 nm and dilutions thereof did not resist UV irradiance. Only broth cultures with critical biomass replicated the UV resistance previously demonstrated by the streak method on agar plates. It was established that UV irradiation (302 nm, 70 W/m^2^) of bacteria for 15–60 min approximates to dilution of bacteria culture in the order of 10^−7^ for control populations **N**_**0**_ averaging between 1.5 x 10^7^ and 2.5 x 10^7 ^CFU.

Methanolic extracts (MEs, 0.5% v/v) in aqueous acetic acid (0.2%) did not show absorption at 334 nm indicative of MAAs, but instead they were bactericidal. All bacterial MEs showed UV absorption in the region of absorbance of DNA (260 nm).

### 3.3 UV survival kinetics of the UV resistant *B. licheniformis*

**Figure 3:**
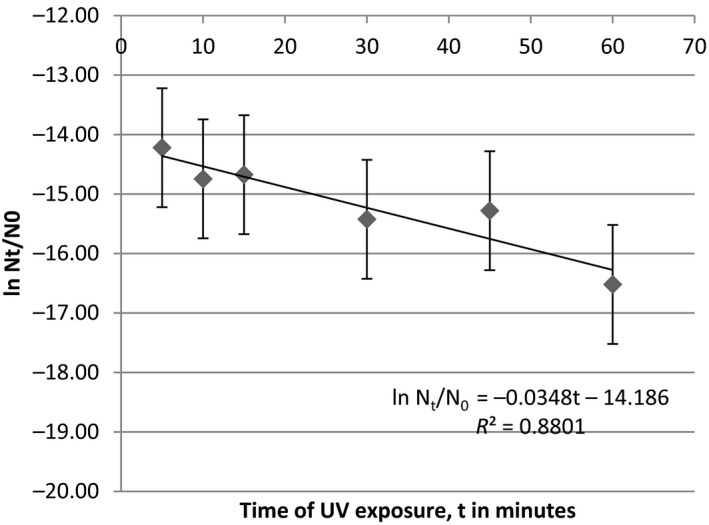
A graph for estimating the population of *B. licheniformis* following UV irradiance

**Figure 4:**
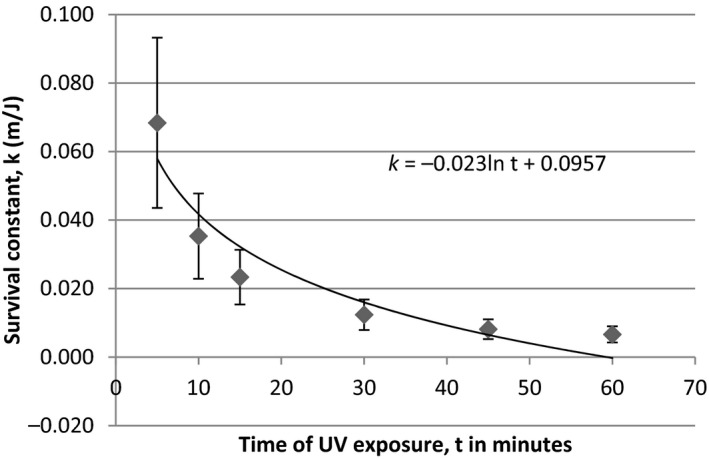
A survival curve for *B. licheniformis* under UV radiation. As the time of exposure increases, there is less variation. The survival constant *k* values are dependent on the total fluence **H** in the equation $k=(H\left(e^{-t}-1\right){\text{ln}\;}_{t}/N_{0}$ . **H** itself is a function of the absolute transmittance **T**, the bandwidth of radiation **b** (nm), the fluence **H**_**0**_ (W/m^2^) and time ***t*** (min)

Data for survival populations of *B. licheniformis* were consistent with first‐order decay trends showing slopes of 0.033 (*R* ^2 ^= 0.88) (Figure**** 3). The survival constant **k** was estimated, using the equation in Figure**** 4. The size of colonies of bacteria increased after UV irradiance for both Gram‐positive and Gram‐negative bacteria. *B. licheniformis* aggregated themselves to increase their colony size during UV irradiance (see supporting data). However, *Escherichia coli* cells have been known to form long filaments as a result of UV irradiance (Kantor & Deering, 1966), this is the first attempt to observe increases in the sizes of colonies of bacteria subjected to UV irradiance. The survival data for *B. licheniformis* unequivocally showed that increases in UV exposure time reduce error margins of the survival constants (Figure**** 4). These results have relevance to germicidal UV irradiance of air, food, and water for sterilization. It is unclear how safe this technique is especially as UV resistant bacteria begin to grow after 48 hr.

### 3.4 *Bacillus licheniformis* aqueous extracts and benzophenone‐3 UV irradiance

**Figure 5:**
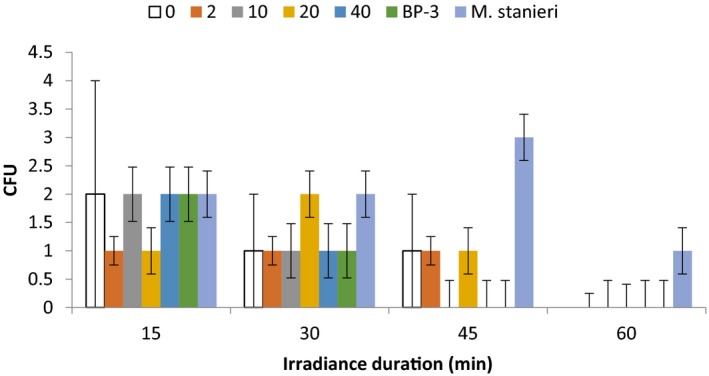
A graph showing the effect of concentrations of *B. licheniformis* AEs on the number of resistant colonies in CFU upon UV irradiance at 15, 30, 45, and 60 min, respectively. The results are the means of three independent experiments. The graph shows that at 60 min, no colonies were observed except for *M. stanieri*. Colonies were observed at lower times of irradiance below 45 min

Aqueous extracts of *B. licheniformis* were investigated for photo‐protective effects on *B. licheniformis* and the less resistant *M. stanieri*. *B. licheniformis* aqueous extracts (AEs) comprised of reconstituted freeze‐dried supernatants of bacteria in sterilized milli Q water at 0, 2, 10, 20, and 40 mg/ml (Figure**** 5). The control group, comprising unirradiated *B. licheniformis* showed growth under all treatments as expected.

Figure**** 5 shows that 0 mg/ml negative control and 20 mg/ml do not have definite patterns for UV irradiance but survived UV irradiation for up to 45 min. However, 10 mg/ml, 40 mg/ml and the positive BP‐3 control had similar pattern in which CFU values decreased to half from 2 to 1 at *t* = 15 and *t* = 30 min exposure and eventually disappearing altogether at *t* = 60 min. 2 mg/ml consistently had 1 CFU at *t* = 15, *t* = 30 and *t* = 45 min and recording zero CFU at *t* = 60 min.

A two‐way analysis of variance (ANOVA) test between AEs treatment and UV irradiance time was not significant (*F *=* *1.12, df = 15, *p *>* *0.05). Subsequently, it was concluded that *B. licheniformis* AEs provided no improved UV survival for the bacteria. Neither did *B. licheniformis* AEs have any protective effect on the UV vulnerable *M. stanieri* strain as conducted against the negative control and a BP‐3 positive control. The BP‐3 positive control (*F *=* *18.95, df = 5, *p *<* *0.05), UV irradiance time (*F *=* *15.79, df = 3, *p *<* *0.05) and the interaction between the two (*F *=* *2.55, df = 15, *p *<* *0.05) were all significant.

## 4 DISCUSSION

The pantropic marine cyanobacterium *M. producens* is ubiquitous in the Western Indian Ocean marine ecosystem (Davies‐Coleman et al., 2003). The isolation of bacteria from the Kenyan *M. producens* is consistent with the nonaxenic nature of the cyanobacterial isolates obtained in this study (Figure 1) and with established literature (Engene et al., 2012). The composition of bacteria isolates varied significantly morphologically and with respect to color at the four sites. The different bacterial phenotypes isolated from *M. producens* suggest that they could have different ecological functions in their association with the cyanobacteria. Pigmentation (e.g., white milky, cream white, deep yellow, and light yellow) was not attributed to location but likely to depend on other factors such as synthesis of secondary metabolites. *Firmicutes/Bacilli* comprised the bulk of isolates followed by β*‐proteobacteria*, α*‐ proteobacteria,* γ*‐proteobacteria*, and *Actinobacteria*, respectively. The occurrence of β*‐proteobacteria* and *Firmicutes* in the Kenyan *M. producens* is similar to previous studies done on *Nodularia* (Salomon et al., 2003; Tuomainen et al., 2006) but contrasts to that by Hube et al., (2009), in which there were no reports of *Firmicutes* and β*‐proteobacteria* isolated from a similar *Nodularia spp*.

The study investigated whether bacteria from *M. producens* have evolved similar adaptations to those of their host cyanobacteria, including UV resistance. It focused on UV resistance of bacteria at the early stages of the stationary phase, before sporulation. Subsequently, the survival kinetics derived here were due to bacterial cells and not spores. Of the 75 bacteria screened for UV tolerance, Gram positive Bacillus strains were the most resistant. *B. licheniformis* from Shimoni exhibited the strongest resistance (302 nm, 70 W/m^2^, 1 hr). Other UV resistant Gram‐positive Bacilli isolates included *B. aerius* (KY646137)*, Paenibacillus pabuli* (KY646125), and *B. cereus* (KY646129, KY646142) from Shimoni and Wasini, respectively. The Gram‐negative *P. stutzeri* (KC660145) and *S. algae sp*. (KC660131) resistance at 45 min was not comparable to *B. licheniformis*. UV irradiance studies on bacteria have mostly been carried out with lower UV exposure times and with sporulation for Gram‐positive bacteria (Mamane‐Gravetz et al., 2005).

Further, the study established that the ability to withstand UV radiation was not dependent on pigmentation. This confers with the hypothesized inability of bacteria to use photo‐protective compounds. However, experiments performed on pigment‐deficient mutants of bacterial isolates showed that inherent pigment conferred increased UV tolerance (Agogué, Joux, Obernosterer, & Lebaron, 2005). The variability could be due to preferential molecular targets in bacteria isolates (Nucleic acids, proteins, and lipids) by the radiation. Bacteria isolates exhibiting an exponential decay and withstanding the maximum dose of radiation with respect to time of exposure were selected and their responses compared. It has been suggested that Gram positive bacteria are better adapted to UV stress because their cell walls screen out a considerable fraction of UV radiation (Jagger, 1985). Among the least resistant bacteria was *Microbacterium koreense* (KY646124) isolated from Mida, a result that is in agreement with observations by Ordoñez, Flores, Dib, Paz, & Farías (2009), demonstrating that cell wall characteristics and G + C content are not the sole determinants of UV resistance.

High optical density values are synonymous with low UV fluence arising from low transmittance. Total fluence (H) was shown to increase with time and decreased with optical density for all the UV resistant bacteria. There are many known density‐dependent regulatory systems in marine bacteria, such as those of virulence and bioluminescence. Survival for *B. licheniformis* (KC660142) was consistent with a first‐order decay curve. Similar relationships have been established for enteric bacteria in natural waters (Darakas, 2002). However, rarely any studies exist for UV mediated survival of bacteria. Survival kinetics and decay curves for enteric bacteria have been constructed from UV irradiance studies on bacteria spores (Mamane‐Gravetz et al., 2005). A factor of H was incorporated into the decay equation to cater for the total fluence emitted by a UV‐VIS radiometer onto the bacteria (Mamane‐Gravetz et al., 2005). The current study utilized this factor but modified it to allow for the estimation of bacteria population surviving UV irradiance over time. For example, half the population of *B. licheniformis* was dead after 0.33 min. A definite pattern was not feasible for *P. stutzeri* and *S. algae sp*. suggesting that these have alternative survival kinetics.

The aqueous extracts and methanolic extract assays aimed to test the effect of *B. licheniformis* secondary metabolites on the UV protection of bacteria. These metabolites had no significant impact on *B. licheniformis* and *M. stanieri* UV resistance. By contrast BP‐3 positive control did exert a protective effect and significantly improved UV resistance to the vulnerable *M. stanieri*. Post hoc analysis established that the significant deviation from the negative control could be attributed to the BP‐3 positive control (*F *=* *18.78, df = 1, *p *<* *0.05). Therefore, BP‐3 had a protective effect over the UV survival of *M. stanieri*, but the *B. licheniformis* AEs did not.

**Figure 6:**
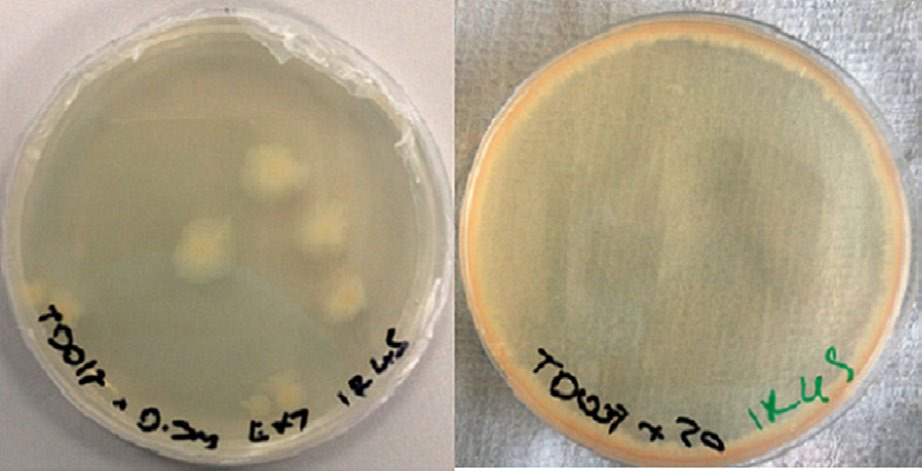
Images of marine agar petri dishes spread with *Bacillus licheniformis* (LHS) and *Marinobacterium stanieri* (RHS). Both dishes were treated with 100 μl of 20 mg/ml of *B. licheniformis* metabolite extract after UV irradiation (302 nm, 70 W/m^2^, 45 min) and incubated (32**°**C, 48 hr). Cells are randomly distributed on the petri dish for *B. licheniformis* and growing at petri dish edges for *M. Stanieri*

*Marinobacterium stanieri* was earlier considered to be vulnerable to UV irradiation (this study). However, it survived up to 1 hr of exposure with colonies almost exclusively located close to or against the edge of the petri dish as opposed to *B. licheniformis* colonies that were randomly distributed across the agar plate (Figure**** 6). Considering this phenomenon was observed only in plates that had been irradiated with UV and not those of the 0 min exposure controls, this clearly suggests an interspecific difference in their response. It is possible that species living within the same consortia have coevolved different mechanisms of UV resistance. We propose the idea that *B. licheniformis* is a ‘UV endurer’, while *M. stanieri* is a ‘UV evader’. ‘UV evaders’ exhibit taxis away from the source of UV radiation and in the case of *M. stanieri*, taxis may have resulted in the accumulation of high enough cell densities at the edge of the dish for some bacteria to survive.

In conclusion, the study has shown that certain bacteria coexisting with *M. producens* have the inherent ability to resist UV radiation for prolonged times. Evolutionary factors and UV resistant molecules may be responsible for resistance. The study demonstrates the need to investigate further the real causes for prolonged UV resistance in certain Gram‐positive and Gram‐negative bacteria. That limited colonies of UV resistant bacteria could only be observed 48 hr after irradiation should be a cause of concern for germicidal irradiation, sterilization of air, food, and water.

## AUTHORS CONTRIBUTIONS

Thomas Dzeha designed the experiments and wrote the manuscript. Dzeha, Constance Nyiro and Dimitris Kardasopoulos performed the laboratory experiments. Joseph Mwafaida and David Mburu supervised the experiments in Kenya; and Michael John Hall and J Grant Burgess contributed towards the manuscript.

## CONFLICT OF INTEREST

The authors declare that there is no conflict of interest.
