# Supplementary material for: UV Resistance of bacteria from the Kenyan Marine cyanobacterium Moorea producens
Source: Microbiologyopen. 2018 Aug 19;8(4):e00697. doi: 10.1002/mbo3.697 (PMC6460272; doi:10.1002/mbo3.697)
Supplement: Supplementary file 1 [file MBO3-8-e00697-s001.docx]

***BACILLUS LICHENIFORMIS* CELLS BEFORE AND AFTER UV IRRADIANCE**


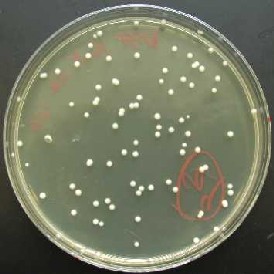


**1. *Bacillus licheniformis* un-irradiated cells**


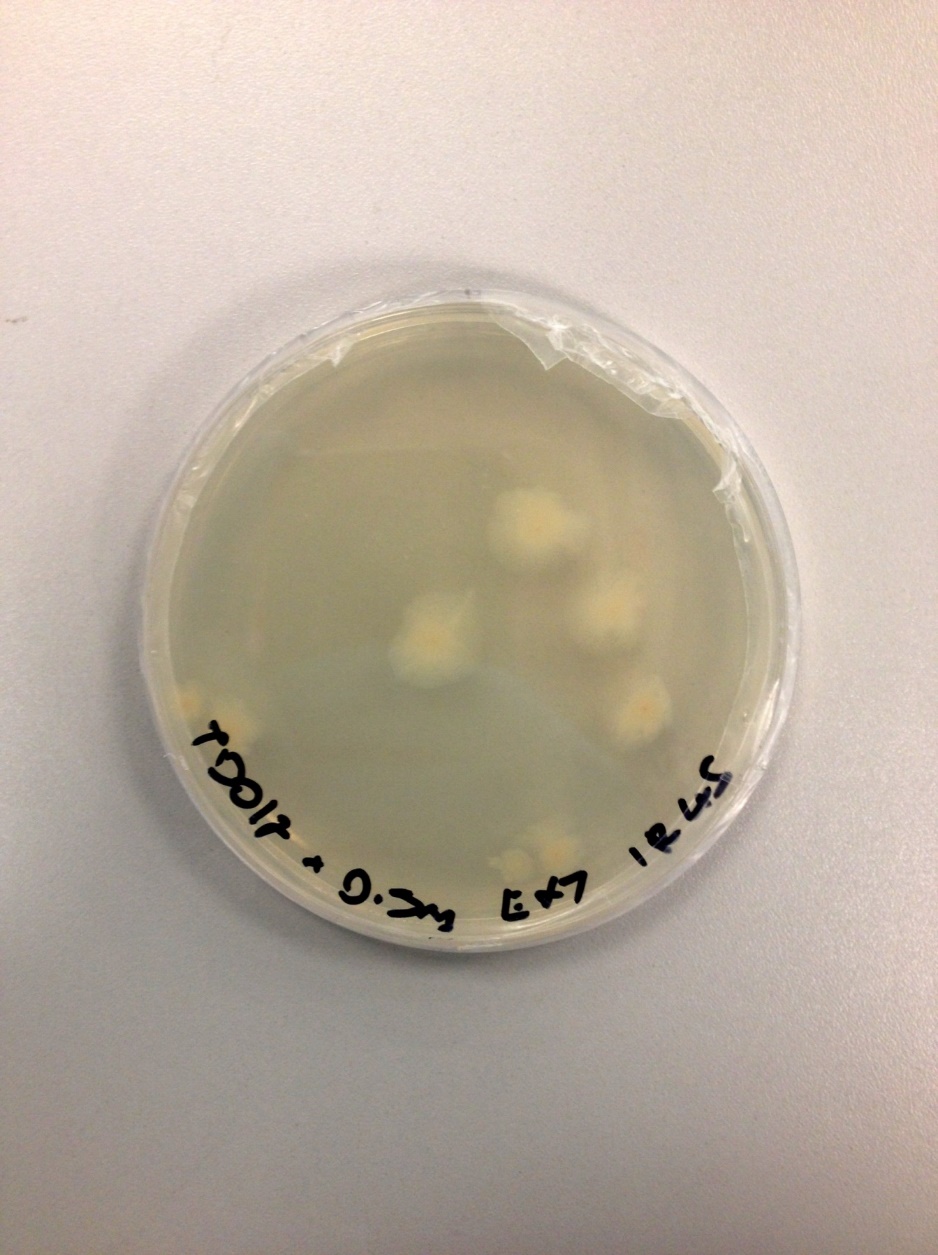


**2. *B. licheniformis* upon irradiance (cells have increased in size)**


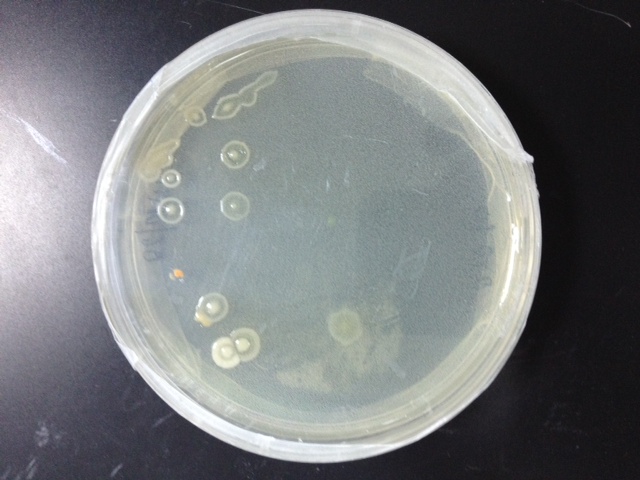


**3*. B. licheniformis* colonies before aggregation**


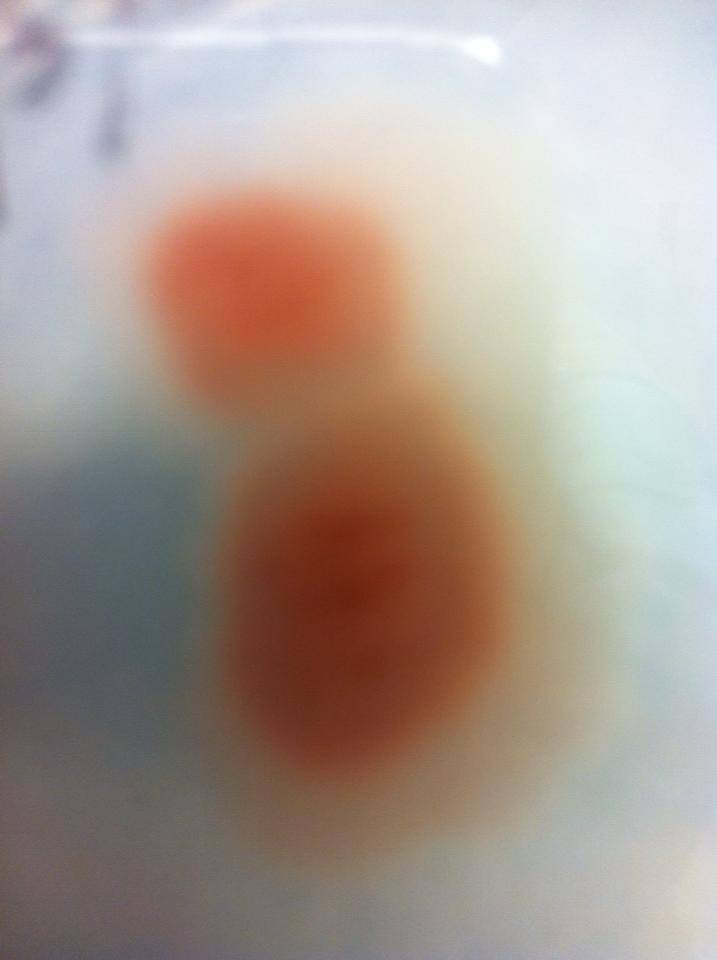


**4. *Bacillus licheniformis* cells aggregating upon UV irradiance**
